# Acute Phase Reaction and Fracture Risk Reduction: Are Gamma‐Delta T Cells and Hypovitaminosis D the Missing Link?

**DOI:** 10.1002/jbmr.4534

**Published:** 2022-03-14

**Authors:** Giovanni Adami, Angelo Fassio, Davide Gatti, Ombretta Viapiana, Maurizio Rossini

**Affiliations:** ^1^ Rheumatology Unit University of Verona Verona Italy

To the Editor:

We read with great interest the paper by Black and colleagues^(^ ^1^ ^)^ on the interaction between acute‐phase reaction (APR) and efficacy for osteoporosis after zoledronic acid. In this post hoc analysis of the Health Outcomes and Reduced Incidence with Zoledronic Acid Once Yearly (HORIZON) trial, women that experienced an APR had a greater fracture risk reduction compared to women that did not experience an APR. However, there is no clear mechanistic explanation to this phenomenon, as pointed out by the authors in the Discussion. One intriguing hypothesis, raised by the authors, regards the well‐known immunological effects of the aminobisphosphonates mediated by the activation of γδ T‐cells. We previously noted that APR after aminobisphosphonate administration was associated with a long‐term decrease in circulating γδ T‐cells and, remarkably, the higher the drop in γδ T‐cells the lower risk of additional APRs after the second zoledronic acid infusion.^(^ ^2^, ^3^ ^)^ In this scenario, aminobisphosphonates might chronically reduce proinflammatory circulating T‐cells; a hypothesis that is supported by several clinical experiences. As a matter of fact, aminobisphosphonate use has been associated with lower risk of severe pulmonary infections^(^ ^4^ ^)^ and higher chance of discontinuation in polymyalgia rheumatica.^(^ ^5^ ^)^ Therefore, because the subjects with prominent inflammation have been shown to have higher fracture incidence,^(^ ^6^ ^)^ zoledronic acid might be beneficial in terms of fracture risk reduction through long‐term anti‐inflammatory action, which is pronounced in patients with APR after infusion. Nonetheless, there is at least another hypothesis possibly explaining the intriguing result by Black and colleagues.^(^ ^1^ ^)^ Indeed, vitamin D baseline status, which is negatively associated with both APR^(^ ^7^ ^)^ and fracture risk, was not considered as a potential confounder in the multivariable analysis. In this case, women with vitamin D deficiency at baseline were at higher risk of experiencing an APR and might had benefited more in terms of fracture risk reduction.

## Disclosures

All authors state that they have no potential conflict of interest related to the present paper.

## Conflict of Interest

GA, AF, DG, OV, and MR declare that they have no conflict of interest related to the present work. All authors have completed the ICMJE uniform disclosure form at www.icmje.org/coi_disclosure.pdf (available on request from the corresponding author) and declare no conflicts of interest.

## Author Contributions

**Angelo Fassio:** Writing – review and editing. **Davide Gatti:** Writing – review and editing. **Ombretta Viapiana:** Writing – review and editing. **Maurizio Rossini:** Conceptualization; supervision; validation; writing – original draft; writing – review and editing.
